# Associations Between Social Media Usage, Physical Activity and Flourishing in Rural University Students

**DOI:** 10.13023/jah.0704.08

**Published:** 2025-12-01

**Authors:** Anna Dysart, Minu Sara Thomas

**Affiliations:** Western Carolina University; Western Carolina University

**Keywords:** Appalachia, college students, flourishing, health, physical activity, social media

## Abstract

**Introduction:**

Most adolescents and young adults report significant use of social media.^1^–^3^ However, there is limited research on the effect of social media use, specifically on physical activity and flourishing.

**Purpose:**

This pilot research study examines the correlation between social media usage and the physical activity and flourishing levels of college students at a rural university in Appalachia.

**Methods:**

The authors conducted a survey including the validated Secure Flourishing Index and Stanford Leisure-Time Activity Categorical Item, containing questions on social media use and demographics. Spearman’s rho was used for nonparametric correlations. Pearson’s correlation was used to determine the correlation of flourishing with physical activity levels.

**Results:**

Time spent on Threads per login was positively associated with higher physical activity (pppp <0.001). Implications: Social media use, both frequency and time spent on the platforms, can impact how college students attending a rural university flourish and participate in physical activity. Continued research in this area should investigate the content on social media for its impact on different domains of flourishing including physical and mental health.

## INTRODUCTION

Most adolescents and young adults report significant use of social media.^1^–^3^ However, there is limited research on the effect of social media use specifically on physical activity and flourishing. Flourishing is defined as the sustained sense of overall well-being and health.^12^ When looking at social media use in college students, a fuller picture of how physical activity, flourishing, and social media may all interact are needed. Research should be done in more rural and smaller sized universities. This pilot research study examines the correlation between social media usage and the physical activity and flourishing levels of college students at a rural university in Appalachia.

## METHODS

Participants were students from a rural university in the Appalachian Region. Recruitment was conducted from April – October 2024. The students were asked about their personal flourishing using the validated Secure Flourishing Index (SFI).^12^

Respondents also self-reported their demographic information. The survey included the use of the Stanford Leisure-Time Activity Categorical Item (L-Cat), which has been validated for use as a measure of the individual meeting the Physical Activity Guidelines for Americans. Participants answered questions on their type of social media use and how long they spent on each social media when they logged-in.

All participants signed an informed consent form before joining the study. The study protocol was approved by the Western Carolina University’s Institutional Review Board (2024-02-16-01). All surveys were collected via Qualtrics (Qualtrics.com, Provo, UT). Statistics were completed for all information using SPSS, version 29.0.2.0 (SPSS Inc., Chicago, IL, USA).

## RESULTS

Seventy-three students completed all parts of the survey and were subsequently included in the data analysis. Participant demographics follow the trend of the university demographics (Table 1).^13^ Instagram was the most frequently used platform, with 93.3% of participants reporting an account. A subset of participants (n=7, 9.3%) reported using all social media platforms. Daily logins were standard among Instagram and TikTok users, with 72.9% and 82% of respondents logging in 6–7 days per week, respectively. TikTok users spent the most time per session, with 56% (28 of 50 users) spending more than 30 minutes per login. The primary reasons for using social media included entertainment (90.7%), addressing boredom (81.3%), and staying informed about trends (56%).

Time spent on Threads per login was positively associated with higher physical activity levels (*p* <0.05) (Table 2). Participation in physical activity significantly correlated with higher flourishing scores (*p* <0.01). Frequent Instagram usage and longer time spent per session on X (formerly Twitter) were positively correlated with flourishing scores (*p* <0.05). Higher Facebook usage frequency was negatively associated with physical activity levels (*p* <0.001).

## DISCUSSION

Physical activity has been correlated with flourishing and quality of life in multiple studies,^10^ and this continues to be true in the findings of this study. Interestingly, a respondent to the survey specifically called out using social media for fitness and nutrition trends. Consuming fitness content on social media may encourage increased physical activity and flourishing. More research is needed on the content type viewed most frequently on each platform and its relationship with physical activity and flourishing in this population.

Social media engagement was diverse among respondents, and only a small percentage used all the platforms studied. This suggests that in order to reach a broad audience, any health promotion activities on social media need to be pushed out to multiple different platforms. Additionally, the findings suggest platform-specific differences in the relationship between social media use, psychological well-being, and physical activity behaviors. Any health promotion activities would need to be adapted to each platform, as well as to each target population.

This pilot study provides valuable preliminary insights into the associations between social media usage, flourishing, and physical activity, serving as a foundation for future research on digital health interventions in an underrepresented population. The accessible study design facilitated participation, resulting in robust data collection. Future studies should aim to address these limitations by incorporating larger and more diverse populations, adopting longitudinal designs, and analyzing social media content to understand its impact on well-being and behavior better.

## IMPLICATIONS

Due to the rural Appalachian geography around the university, there is limited infrastructure (greenways, sidewalks, bike lanes, etc.) to support physical activity promotion in the community for individuals living off campus. While there is a campus recreation center that students can go to, students have to pay for fitness classes. Additionally, the closest town with more than 3,000 residents is a 35-minute drive. This may lead to more time spent on social media as an entertainment option.

Correlation of time on X (formerly Twitter) with higher flourishing scores, as well as the amount of time on Threads being correlated with higher physical activity, may be due to the content consumed on these platforms during that time. Threads and X also have similar content structure, with them both being more text-based than TikTok or Instagram, which may contribute to differences seen between those platforms.

This pilot study expands the knowledge of how social media can impact rural college students, which are a currently underrepresented demographic in the literature. Social media use, both frequency and time spent on the platforms, can impact how college students attending a rural university flourish and participate in physical activity. Continued research in this area should investigate the content on social media for its impact on different domains of flourishing including physical and mental health.

**SUMMARY BOX**

**What is already known about this topic?**

Social media use is high in adolescents and young adults and can affect physical activity and mental health. Physical and mental health are part of flourishing (i.e., overall well-being).

**What is added by this report?**

This pilot study adds information on how social media use affects the flourishing of the rural Appalachian student population.

**What are the implications for future research?**

Future studies should aim to address these limitations by incorporating larger and more diverse populations, adopting longitudinal designs, and analyzing social media content to understand its impact on well-being and behavior better.

## Figures and Tables

**Table 1:** Demographic Comparison of Survey Respondents and University Student Population

	Survey Respondents	University Demographics
**Gender:**		
Female	n=49 (67.1%)	59%
Male	n=23 (31.5%)	41%
Nonbinary	n=1 (1.4%)	Not reported
**Race:**		
Native American	n=0	0.9%
Asian	n=5 (6.8%)	1.3%
Black	n=4 (5.5%)	6.8%
Native Hawaiian or Pacific Islander	n=0	0.1%
White	n=63 (86.3%)	75.3%
Do not wish to disclose/Unknown	n=1 (1.4%)	2.9%
**Current Academic Classification**		
Freshman	n=10 (13.7%)	
Sophomore	n=10 (13.7%)	
Junior	n= 24 (32.9%)	
Senior	n=9 (12.3%)	
Grad	n=18 (24.7%)	
Do not wish to disclose	n=2 (2.7%)	
**Social Media Accounts**		
X (formerly Twitter)	n= 26 (34.7%)	
Instagram	n=70 (93.3%)	
Facebook	n=49 (65.3%)	
Threads	n=13 (17.3%)	
TikTok	n=50 (66.7%)	
**Why do you use social media?**		
Peer pressure	n=2 (2.7%)	
Keep up with trends	n=42 (56%)	
Boredom	n= 61 (81.3%)	
Entertainment	n=68 (90.7%)	
It’s my job/marketing	n=10 (13.3%)	
News/Information	n=35 (46.7%)	
Other	n=10 (13.3%)	

**Table 2:** Social Media Habits and Correlations with Physical Activity and Flourishing

Type of Social Media	Flourishing	Physical Activity Level
**Instagram**		
Frequency	*p* =0.028 (r=0.262)	*p* =0.293
Time Spent per Log-on	*p* =0.060	*p* =0.910
**Facebook**		
Frequency	*p* =0.218	*p* <0.001 (r = −0.545)
Time Spent per Log-on	*p* =0.251	*p* =0.105
**Twitter/X**		
Frequency	*p* =0.696	*p* =0.002
Time Spent per Log-on	*p* =0.033	*p* =0.901
**Threads**		
Frequency	*p* =0.487	*p* =0.690
Time Spent per Log-on	*p* =0.071	*p* =0.033 (r =0.592)
**TikTok**		
Frequency	*p* =0.506	*p* =0.184
Time Spent per Log-on	*p* =0.081	*p* =0.539
